# Contrast ureteropyelography in theatre: standardised flowchart reporting

**DOI:** 10.1308/003588412X13171221500385

**Published:** 2012-07

**Authors:** MA Harris, T Marsh, A Llewellyn, A West, G Naisby, BDR Gowda

**Affiliations:** ^1^Salford Royal NHS Foundation Trust; ^2^South Tees Hospitals NHS Foundation Trust

**Keywords:** Urography, Ureter, Kidney, Standards, Ureteroscopy

## Abstract

**INTRODUCTION:**

Urologists perform retrograde contrast studies of the ureters and pelvicalyceal systems in the operating theatre, both for diagnostic purposes and to guide instrumentation. We describe the development of a set of guidelines that aim to standardise the diagnostic quality of these studies and to reduce radiation dose to the patient and theatre staff. The guidelines incorporate a reporting template that allows a urologist’s written report to be made available on the picture archiving and communication system (PACS) for subsequent multidisciplinary review.

**METHODS:**

Three cycles of audit were conducted to assess the implementation of the guidelines. An independent reviewer rated image quality and screening times. During the audit cycle, the presentation of the guidelines was honed. The end product is a flowchart and reporting template for use by urologists in the operating theatre.

**RESULTS:**

Phase 1 of the audit included 63 studies, phase 2 included 42 studies and phase 3 included 46 studies. The results demonstrate significant improvements in the number of good quality studies and in the recording of control, contrast and post-procedure images. The mean screening time decreased from 5.0 minutes in phase 1 to 3.2 minutes in phase 3. In phase 3, when in-theatre reporting of the studies by the urologist was added, the handwritten report was scanned in and made available on PACS in 43 of 46 cases (93%).

**CONCLUSIONS:**

Introduction of guidelines improved retrograde contrast study quality and reduced screening times. A system has been developed to store appropriate pictures and a urologist’s report of the study on PACS.

Although retrograde ureteropyelography (RUP) has lost its prominence as a primary diagnostic modality in urology, contrast studies are frequently used in theatre to guide instrumentation. Most retrograde contrast studies are observed and interpreted ‘on the spot’ by the urologist, with many patients undergoing ureteroscopy in theatre at the same time. Frequently, the retrograde studies are performed without senior supervision. Before introducing guidelines, subsequent review of the images often demonstrated that appropriate images were not stored, resulting in incomplete visualisation of the urinary tract and focal areas of abnormality.

The guidelines were developed to improve teamwork between urologists and radiographers in theatre and to help them acquire diagnostic-quality images consistently. When stored on the picture archiving and communication system (PACS), high quality images can provide valuable information in multidisciplinary team (MDT) meetings and aid treatment planning.

## Development of standard technique for retrograde contrast studies

The indications for RUP were separated into several categories with the technique adjusted according to the aim of the procedure. These were summarised into a quick reference guide (Table 1). They were accepted as the standard following the initial cycle of audit (phase 1), which documented baseline performance. After the introduction of the guidelines, we conducted a phase 2 audit to test compliance.

**Table 1:** Quick reference guide to retrograde ureteropyelography in operating theatre

Type of retrograde study	Control images over kidney, upper, middle and lower ureter	Injection of contrast	Additional images	Comments
Emergency stenting	Yes, unless recent imaging	Minimal to allow positioning the stent	Post-procedure images unless post-operative KUB is planned	Aimed at unblocking the obstructed urinary tract, preferably by bypassing the obstruction with ureteric catheter initially
Elective diagnostic retrograde study	Control film of the KUB region (KUB in radiology department or may need 3 exposures)	Contrast study capturing entire length of the ureter (may need 2–3 exposures) with sequential images while injecting the contrast agent		Includes elective insertion of stent, first time PCNL and ureteroscopy
Elective treatment of ureteric / renal calculi	Pre-treatment stone position should be shown	Contrast injection to guide the instrumentation	Post-procedure images unless post-operative KUB is planned	Includes repeat PCNL and ureteroscopy
Endourological management of upper urinary tract tumours	Control over the location of the lesion	Contrast images delineate the lesion and the entire ureter	Post-procedure images to document treatment effect. Save images in cases of stenting unless post-operative KUB is planned.	
Elective change of stent	Controls to document stent encrustation if present	Contrast injection to facilitate stent positioning	Post-procedure images unless post-operative KUB is planned	

KUB = kidney, ureters and bladder x-ray; PCNL = percutaneous nephrolithotomy

**Figure 1:**
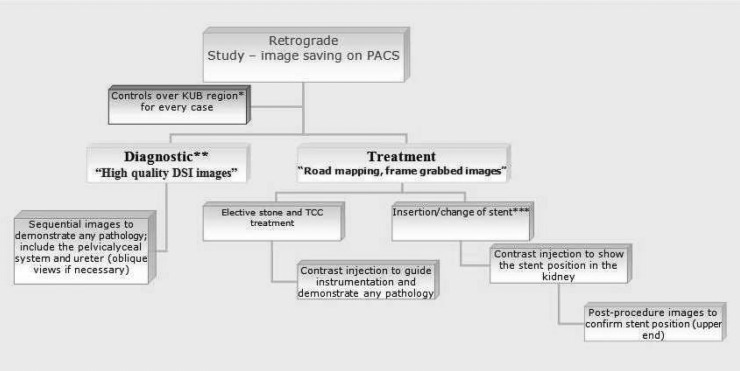
Retrograde ureteropyelography technique flowchart

To improve the acceptability and user-friendliness of the guidelines, a flowchart (Fig 1) and urologist reporting template were designed that included information on the type of retrograde study, its findings and a space for free text. The report was scanned into PACS to be available during multidisciplinary review.

PACS = picture archiving and communication system; KUB = kidney, ureters and bladder x-ray; DSI = digital spot images; TCC = transitional cell carcinoma

*KUb is performed over kidney, ureter and bladder on the affected side.

**diagnostic retrograde study must include some dSI with higher resolution and radiation exposure.

***Emergency stent insertion requires minimal contrast injection after decompressing the kidney by aspiration.

Please transfer all images taken to PACS.

The phase 3 audit re-tested compliance following the introduction of the flowchart and template.

## Methods

Prospective data were collected on all intra-operative RUP contrast studies performed in a major urology unit at a university hospital over three two-month study periods. This included periods before and after the introduction of the new guidelines. All operators were asked to clearly record the procedure type on the x-ray request card (diagnostic or therapeutic; elective or emergency).

The images stored on PACS were assessed by a third party observer (AL), who recorded: the number of stored images; control images taken over pelvis, sacroiliac joints, upper ureters and kidneys; the presence of contrast in pelvic ureters, over sacroiliac joints, mid-ureters and kidneys; the degree of collecting system filling (good distension, extravasation/backflow, underfilling); post-procedure images; the presence of bubbles; and the duration of screening.

Each study was compared to the guideline standard and rated as compliant or non-compliant for control, contrast and post-procedure images. The quality of the studies was also rated as one, two or three stars depending on the degree of collecting system filling and the presence of bubbles. We aimed to achieve 80% compliance with the guidelines as our audit standard.

Statistical analysis was performed with the chi-square test, Fisher’s exact test and Student’s t-test using SPSS® version 11 (SPSS Inc, Chicago, IL, US).

## Results

Phase 1 analysed 63 studies, phase 2 analysed 42 studies and phase 3 analysed 46 studies (Table 2). The number of good quality studies (‘three stars’) increased from 67% before the introduction of the guidelines to 74% at the phase 3 audit (chi-square test, *p*<0.05).

**Table 2:** Compliance rate with the guidelines

Parameter	Phase 1	Phase 2	Phase 3
Total number of retrograde studies	63	42	46
Good quality studies (‘three stars’)	42 (67%)	32 (76%)	34 (74%)
Control image recording according to guidelines	25 (40%)	28 (67%)	35 (76%)
Contrast image recording according to guidelines	36 (57%)	33 (76%)	42 (91%)
Post-procedure image recording if stent is inserted	19/26 (73%)	20/21 (95%)	22/22 (100%)

Control image recording improved significantly for both diagnostic and treatment studies (73% and 80%) in phase 3, approaching the audit standard. Contrast image recording also improved significantly, with the treatment category achieving the audit standard (80%) and the diagnostic category improving to 65%. Post-procedure images to confirm the position of the stent were taken in all cases in phase 3 of the audit, a significant improvement compared with the other two phases.

The screening time was shorter in the phase 3 audit and there was an increase in the number of acquired images (Table 3). In this audit phase, a report by the urologist was available on PACS in 43 cases (93%) and the majority of the urologists found the practice acceptable.

**Table 3:** Radiation exposure indicators

Indicator	Phase 1	Phase 2	Phase 3	*p*-value (ANOVA)
Mean screening time (minutes)	5.0	4.5	3.2	**0.04**
Mean number of acquired images	7.6	7.8	9.7	**0.02**

## Discussion

In 1906 Voelcher and von Lichtenberg were the first to successfully visualise the ureter and renal pelvis using a colloidal suspension of silver. Although still a valuable technique, retrograde ureteropyelography is used less frequently since the advent of intravenous pyelography, ultrasonography, computed tomography and magnetic resonance imaging.^1,2^

*Campbell-Walsh Urology* recommends the use of diagnostic RUP when assessment of the upper urinary tract is required during the evaluation of haematuria.^3^ Other indications are persistent filling defects of the ureter or renal collecting system, unexplained positive urinary cytology collected from the upper urinary tract and fistulas or obstructions involving the ureter. The textbook also summarises patient preparation, types of catheters used, technique of introduction of the catheter into the ureter, injection of contrast material and types of images to be taken. However, there are no guidelines for the recording of retrograde studies performed in theatre.

We have found only a brief description of technique in the radiological literature.^4,5^ RJ Zagoria recommends that for a study result to be considered normal, the entire collecting system must be demonstrated and if a ureteric filling defect is present, the entire length of the ureter should be demonstrated.^6^ Indeed, the procedure is now only rarely performed by a radiologist. Other non-invasive techniques such as multi-detector computed tomography and magnetic resonance imaging have replaced RUP for most indications outside the operating theatre.^2,7^

Semi-rigid and flexible ureterorenoscopic techniques have rendered the upper urinary tract easily accessible to direct visualisation. Unfortunately, these are invasive procedures with attendant risks of serious complications such as ureteric injury. In some cases, ureteric stricture may not allow passage of even the smallest diameter instrument. RUP is a crucial diagnostic alternative in these cases and a retrograde contrast study is commonly carried out prior to instrumentation of the upper urinary tract by the urologist.

To our knowledge, this study is the first modern attempt to standardise the performance and reporting of retrograde contrast studies in theatre by urologists, with the aim of allowing subsequent review of diagnostic-quality images by a radiologist in the MDT setting. Retrograde imaging requires good teamwork between radiographers and urologists. Ideally, RUP should be performed in the presence of a senior clinician. However, it is not always possible so the proposed guidelines are especially useful in cases when the task is delegated to a trainee.

These guidelines and reporting form have been designed to be user friendly. The last phase of our audit demonstrated improved quality of retrograde studies and reduced screening time in theatre, which also might be the result of increased experience of the technique and reflective learning during the audit process rather than an effect of the guidelines. Other intended benefits include the need for fewer repeat retrograde studies or other follow-up imaging studies. Prior to the guidelines being introduced, not all the images captured during RUP at our unit were stored on PACS. Now, however, a representative set of images is stored as a matter of course for all our patients as the team is aware which images are important for subsequent MDT review.

## Conclusions

This guideline introduction and audit shows that a new system of flowchart reporting improved the quality of retrograde studies performed in operating theatres at our hospital. The guidelines responded to the training needs of junior urologists and radiographers by introducing a benchmark and led to a consistent reduction in the radiation exposure of patients and staff as well as improved knowledge of the technique by the MDT.
